# Identification of novel enriched recurrent chimeric *COL7A1-UCN2* in human laryngeal cancer samples using deep sequencing

**DOI:** 10.1186/s12885-018-4161-8

**Published:** 2018-03-02

**Authors:** Ye Tao, Neil Gross, Xiaojiao Fan, Jianming Yang, Maikun Teng, Xu Li, Guojun Li, Yang Zhang, Zhigang Huang

**Affiliations:** 1 Department of Otolaryngology-Head and Neck Surgery, Key Laboratory of Otolaryngology Head and Neck Surgery, Beijing Tongren Hospital, Capital Medical University, Beijing, 100730 China; 2 Department of Head and Neck Surgery, The University of Texas MD Anderson Cancer Center, Houston, TX 77030 USA; 3 Hefei National Laboratory for Physical Sciences at Microscale, Innovation Centre for Cell Signaling Network, School of Life Science, University of Science and Technology of China, Hefei, Anhui 230026 People’s Republic of China; 4 Department of Otolaryngology-Head and Neck Surgery, the Second Affiliated Hospital of Anhui Medical University, Hefei, 230601 China

**Keywords:** Laryngeal cancer, Transcription-induced chimera, Gene fusion, *COL7A1*, *UCN2*

## Abstract

**Background:**

As hybrid RNAs, transcription-induced chimeras (TICs) may have tumor-promoting properties, and some specific chimeras have become important diagnostic markers and therapeutic targets for cancer.

**Methods:**

We examined 23 paired laryngeal cancer (LC) tissues and adjacent normal mucous membrane tissue samples (ANMMTs). Three of these pairs were used for comparative transcriptomic analysis using high-throughput sequencing. Furthermore, we used real-time polymerase chain reaction (RT-PCR) for further validation in 20 samples. The Kaplan-Meier method and Cox regression model were used for the survival analysis.

**Results:**

We identified 87 tumor-related TICs and found that *COL7A1-UCN2* had the highest frequency in LC tissues (13/23; 56.5%), whereas none of the ANMMTs were positive (0/23; *p* < 0.0001). *COL7A1-UCN2*, generated via alternative splicing in LC tissue cancer cells, had disrupted coding regions, but it down-regulated the mRNA expression of *COL7A1* and *UCN2.* Both *COL7A1* and *UCN2* were down-expressed in LC tissues as compared to their paired ANMMTs. The *COL7A1*:β-actin ratio in *COL7A1-UCN2*-positive LC samples was significantly lower than that in *COL7A1-UCN2*-negative samples (*p* = 0.019). Likewise, the *UCN2*:β-actin ratio was also decreased (*p* = 0.21). Furthermore, *COL7A1-UCN2* positivity was significantly associated with the overall survival of LC patients (*p* = 0.032; HR, 13.2 [95%CI, 1.2–149.5]).

**Conclusion:**

LC cells were enriched in the recurrent chimera *COL7A1-UCN2*, which potentially affected cancer stem cell transition, promoted epithelial-mesenchymal transition in LC, and resulted in poorer prognoses.

**Electronic supplementary material:**

The online version of this article (10.1186/s12885-018-4161-8) contains supplementary material, which is available to authorized users.

## Background

There were an estimated 26,400 new cases of and 3620 deaths from laryngeal cancer in China in 2015 [1]. Like other carcinomas of the respiratory system, carcinogen exposure via tobacco smoke causes DNA damage, and the accumulation of this DNA damage can alter genetic and epigenetic regulatory functions and thereby transform normal cells into cancer cells [2, 3]. This cell transformation usually takes multiple steps to complete, and it is affected by the sensitivity of the individual and the degree of damage [4]. This process is called tumorigenesis [5].

Tumorigenesis often presents with chromosomal and DNA abnormalities, and one common chromosomal rearrangement is gene fusion [6]. Some specific gene fusions have become important diagnostic markers of and therapeutic targets in cancer over the past several decades [7]. These chimeric products are often associated with neoplastic behavior [7, 8]. Typically, the *BCR-ABL1* fusion gene is rearranged via the t(8;14)(q24;q32) translocation in Burkitt lymphoma cells. This rearrangement is caused by this gene’s juxtaposition with regulatory elements of the immunoglobulin heavy chain gene at 14q32, where the *MYC* gene is constitutively activated due to its expression, which is driven by immunoglobulin enhancers [7, 9]. Other fusion genes, including *PRCC-TFE3* in papillary renal cell carcinoma [10], *PAX8-PPARG* in follicular thyroid carcinoma [11], *FUS-CREB3L2* in soft tissue sarcoma [12], and *TMPRSS2-ETS* in prostate cancer [13], have gradually been identified with various potential gene regulation mechanisms.

As in the fusion of two DNA genes, the two adjacent RNA genes, which are in the same orientation and are usually transcribed independently, are occasionally transcribed into a single fused RNA sequence. The various splicing mechanisms involved in such a transcription include RNA editing, alternative splicing (AS), *trans*-splicing, alternative transcription start sites, and alternative polyadenylation transcription termination sites [14–17]. This single fused RNA sequence is called a transcription-induced chimera (TIC) [14]. Unlike a single transcript that can be translated into various proteins in prokaryotes, TICs usually do not produce chimeric proteins or independent transcripts. Instead, they have tumor-promoting properties as hybrid RNAs [14]. For example, the expression of the chimeric transcript *HBx-LINE1* was associated with hepatocellular carcinoma development and correlated with poor survival [18]. Also, the chimeric transcript *SLC45A3-ELK4*, generated by *cis*-splicing between the adjacent *SLC45A3* and *ELK4* genes, did not involve DNA rearrangements or *trans*-splicing and could augment prostate cancer cell proliferation [19].

In comprehensively analyzing novel TICs in transcriptomes in LC cells using a paired-end strategy for RNA deep sequencing, we found that *COL7A1-*urocortin 2 (*UCN2*) is a novel TIC. We could not elucidate the intrinsic genetic and epigenetic mechanism responsible for *COL7A1-UCN2* generation; however, both the *COL7A1* and *UCN2* genes had explicit suppressor roles in tumor regulation, specifically the regulation of the epithelial-mesenchymal transition (EMT) [20–22]. Therefore, we hypothesized that *COL7A1-UCN2* may down-regulate the mRNA expression of both *COL7A1* and *UCN2* in LC tissues and that such down-regulation may promote tumor invasion via EMT regulation. Furthermore, we also speculate that *COL7A1-UCN2* generation can reflect the degree of DNA damages and that this TIC positivity may be associated with LC prognosis.

## Methods

### Patients and tissue samples

The Institutional Review Board approval for this laryngeal cancer research project (No. TRECKY 2009–33; Date: Jan, 2009) was obtained from the Beijing Tongren Hospital of Capital Medical University. A total of 23 patients who underwent surgery for pathologically confirmed LC from 2009 to 2016 were enrolled in this study. All patients received and signed a written informed consent. These patients had archived tumor specimens and data available, with a minimum of 36 months of cancer-free or censored-death follow-up after surgery. The follow up was completed through monitoring of their medical records or conducting telephone interviews. To confirm the diagnosis, the tumors’ histological classifications and differentiation were defined based on the 1999 World Health Organization’s histological classification standards for LC. Tumor staging was carried out using the 2009 TNM staging criteria of the Union for International Cancer Control. Clinicopathological data were available for all 23 patients (Table 1).

**Table 1:** Correlation of *COL7A1-UCN2* expression with LC clinical characteristics

Characteristics	Cases (%)	*COL7A1-UCN2* mRNA expression		
		mRNA positive (+)	mRNA negative (−)	*p*
		N, %	N, %	
Age (years)				0.685
≥ 60	15 (65.2)	9 (39.1)	6 (26.1)	
< 60	8 (34.8)	4 (17.4)	4 (17.4)	
Gender				1.000
Male	20 (87.0)	11 (47.8)	9 (39.1)	
Female	3 (13.0)	2 (8.7)	1 (4.3)	
Tumor stage				0.221
I─II	8 (34.8)	3 (13.0)	5 (21.7)	
III─IV	15 (65.2)	10 (43.5)	5 (21.7)	
Differentiation				1.000
Well	4 (17.4)	2 (8.7)	2 (8.7)	
Moderate	16 (69.6)	9 (39.1)	7 (30.4)	
Poor	3 (13.0)	2 (8.7)	1 (4.3)	
LNM				0.685
N0	10 (43.5)	5 (15.6)	5 (15.6)	
N+	13 (56.5)	8 (34.8)	5 (15.6)	
Treatment				0.685
S only	8 (34.8)	4 (17.4)	4 (17.4)	
S + C/X	15 (65.2)	9 (39.1)	6 (26.1)	
Smoking				0.435
Ever	22 (95.7)	13 (56.5)	9 (39.1)	
Never	1 (4.3)	0 (0.00)	1 (4.3)	
Alcohol				1.000
Ever	21 (91.3)	12 (37.5)	9 (39.1)	
Never	2 (8.7)	1 (4.3)	1 (4.3)	

*LNM* lymph node metastasis, *+* positive, *C* chemotherapy, *X* radiation, *S* surgery

All tumor samples contained more than 50% tumor cells and were stored at − 80 °C until use. Paired LC and adjacent normal mucous membrane tissue samples (ANMMTs) were obtained from the 23 patients. Paired samples from three male patients with T4N2aM0 disease and various degrees of differentiation (well, moderately, and poorly differentiated) who were 61–63 years old, smokers, and alcohol drinkers and had undergone total laryngectomy with selective bilateral neck dissection and without preoperative chemotherapy or radiotherapy were prepared for transcriptomic analysis. The paired samples from the remaining 20 patients were used to validate the TIC using real-time polymerase chain reaction (RT-PCR). Adjacent normal tissue samples were obtained at least 5 mm from the tumor margins [23].

### Pathological review

Slides with hematoxylin and eosin staining were used to contain the paired frozen tumor and normal tissue sections. These slides were subjected to pathological examination twice to ensure that tumor tissues carrying high-density cancer foci (> 75%) were used and that the normal tissue samples had no tumor components. All samples were examined and reviewed by two pathologists independently, and disagreements between them were resolved via negotiation.

### Preparation and sequencing of cDNA library

The total RNA was isolated from the fresh tissues using TRIzol reagent (Sigma-Aldrich, Missouri, St. Louis, US) according to the manufacturer’s instructions. Poly(A) mRNA was isolated from the total RNA using beads containing oligo(dT). A fragmentation buffer was used to fragment the purified mRNA. Using these short mRNA fragments as templates, random hexamer primers were applied to synthesize first-strand cDNA. The fragmentation buffer, RNase H, and DNA polymerase I were used to synthesize the second-strand cDNA. Short double-stranded cDNA fragments were purified using a QIA quick PCR extraction kit (Qiagen, Hilden, Germany) and eluted with EB buffer for end repair and the addition of an “A” base. The short fragments were then ligated to Illumina sequencing adaptors (San Diego, CA, U.S.A.). DNA fragments of a selected size were gel-purified and amplified using PCR. The amplified library of fragments was sequenced using an Illumina HiSeq 4000 sequencing machine.

### Raw read filtering

The images of the nucleotides generated by the Illumina HiSeq 4000 sequencing machine were converted into nucleotide sequences using a base-calling pipeline. The raw reads of the nucleotide sequences were saved in FASTQ format. The dirty raw reads were removed before the data analysis. Three removal criteria were used in filtering out dirty raw reads: 1) reads with sequence adapters, 2) reads with more than 2% “N” bases, and 3) low-quality reads. This ensured that clean reads were used for the subsequent mapping to the human genome and transcriptome.

### Reads mapped to the human genome and transcriptome

The Burrows-Wheeler Aligner software program was used to map clean reads to a reference genome, and the Bowtie software program was used to map them to a reference gene. The expression level of each gene was measured via the number of specific fragment reads mapped per kilobase exon model per million reads (RPKM). The formula used for mapping is as follows: $\mathrm{RPKM}=\frac{10^9C}{NL}$. In this formula, *C* stands for the number of fragments specifically mapped to a given gene, *N* stands for the number of fragments specifically mapped to all genes, and *L* stands for the overall length of exons for the given gene. For genes with more than one alternative transcript, the longest transcript was chosen for the calculation of the RPKM. The RPKM calculation avoids the effect of differing gene lengths and sequencing discrepancies. Thus, the differences in the gene’s expressions between samples were directly compared using the RPKM.

### Differentially expressed gene analysis

Differentially expressed genes were identified in the tumor and matched normal tissue samples according to two criteria — a false-discovery rate no greater than 0.001 and a log2 ratio of at least 1. This approach was chosen based on the significance of digital gene expression profiles.

### Fusion of human gene detection

During the read alignment of the short RNA and the reference genome, when the reads were divided into two fragments, only some of them could be aligned. Two-segment alignments could be read to the reference genome using the gene fusion-detection doctrines of the SOAPfuse software program, which can detect gene fusions using span and junction reads [24]. This basic method includes 1) comparing the reads to the reference genome alignment and the transcript**s** to the notes; 2) using the local genome library, which contains an exhaustive algorithm, to construct the fusion site sequence; and 3) retaining highly credible fusion transcripts using a series of filtering means. The requirements for the alignment detection of the divided reads were as follows: a length of at least 8 bp for the shorter read segment and an intron boundary within one of the three canonical bounds (GT-AG, GC-AG, and AT-AC). Regardless of where the intron was derived, the boundaries always should be the same. For the DNA positive strand, for both read segment alignments, a maximum of one mismatch and an unmapped alignment was required. Based on the information on the alignments of the two segments, gene fusion sites identified from the mapping of the human genome and transcriptome were retrieved using a Perl script. A fused gene certainly existed if the fusion site was located at the known exon boundaries of the two genes, with at least one paired-end read supporting it [25–27].

### Detection of alternative splicing (AS)

AS is a fundamental mechanism of the generation of transcript diversity*.* The base-calling pipeline used in this study to detect AS events in the transcriptome cDNA library consisted of two major steps. 1) SOAPsplice (Version 1.1) was used to map the reads to the human reference sequence and report the splice junctions according to the junction reads of the alignments [24]. With SOAPsplice, the default parameters were used as much as possible; three mismatches were set for intact alignments, and no more than one mismatch was set for splicing alignments. 2) Abased on AS mechanisms, both the junctions of splicing [e.g., known splice junctions obtained from the National Center for Biotechnology Information RefSeq database (Bethesda, MD, US)] and the results derived from the mapping were applied for the detection of the four basic AS events: the skipping of exons, sites of alternative 5′ splicing, sites of alternative 3′ splicing, and the retention of introns.

By detecting the four types of AS events, those that occurred in the tumors, rather than in the matched normal tissue, were detected as specifically tumor-related AS events. The AS events that were detected in both LC and ANMMT samples were then filtered. Finally, for each sample, a list of highly reliable tumor-specific AS events was generated.

### Validation of transcriptome cDNA library using RT-PCR

To determine the frequency of *COL7A1-UCN2* and *COL7A1* and *UCN2* mRNA expression, the other 20 paired LC and ANMMT samples were subjected to RT-PCR analysis*.* The primer sequences used for this RT-PCR are listed in Table 2.

**Table 2:** Primer sequences used for RT-PCR in the study

Primers	Sequences
*COL7A1-UCN2*	F: 5’-CGCCAAGAGATGAGTCAGCAC-3’
	R: 5’-GCACTCAGATCTGATATGACCTGC-3’
*COL7A1*	F:5’-CGCCAAGAGATGAGTCAGCAC-3’
	R:5’-CTCTGCAGGTAGGGCAGGGT-3’
*UCN2*	F:5’-ATGACCAGGTGTGCTCTGCTGTTGC-3’
	R: 5’-TCAGCAGTGGCCGACACG-3’
*β-actin*	F:5’-TTGCCGACAGGATGCAGAA-3′
	R:5’-GCCGATCCACACGGAGTACTT-3′

For the cDNA of *COL7A1-UCN2* and *COL7A1*, the PCR conditions were 10 min at 95 °C, 30 cycles of 30 s at 95 °C, 30 s at 62 °C, 90 s at 72 °C, and 10 min at 72 °C. For *UCN2* cDNA, the PCR conditions were 10 min at 95 °C, 30 cycles of 30 s at 95 °C, 30 s at 70 °C, 30 s at 72 °C, and 10 min at 72 °C. β-actin was used as a loading control. The RT-PCR products were analyzed using gel electrophoresis.

Quantitative analysis of PCR products was carried out using a Rotor-Gene 3000 (Corbett Research, Sydney, Australia) and a commercially available SYBR Premix Ex Taq Perfect Real-Time Kit (Takara Biotechnology, Dalian, China), which were used according to the manufacturer’s instructions. The primer sequences used were those described above. The PCR conditions were 30 s at 95 °C, 40 cycles of 5 s at 95 °C, and 30 s at 60 °C. The data were analyzed using the ΔΔCt method, and values were expressed as the fold difference from the housekeeping gene, β-actin.

### Statistical analysis

Data were expressed as means ± standard deviation. Differences between the two groups were examined using Fisher’s exact test (two-sided, *n* < 40) or a paired or unpaired Mann-Whitney *U-*test. The Kaplan-Meier method and Cox regression model were used to perform the overall survival analysis of the 23 patients, who were grouped according to their positivity or negativity for *COL7A1-UCN2*. *P*-values less than 0.05 were considered statistically significant. The data were analyzed using the SPSS 20.0 statistical software program (IBM Corporation, Armonk, NY, USA).

## Results

### Transcriptome sequences in human LC and ANMMT samples

We compared the transcriptome sequences in LC and paired normal tissue samples and identified a series of gene fusions and differentially expressed genes. The RNA sequencing data for the three pairs of LC and ANMMT samples subjected to transcriptomic analysis are listed in Table 3.

**Table 3:** RNA sequencing data for three pairs of LC and ANMMT samples for transcriptomic analysis

Sample		Clean reads	Expressed genes	Expressed Transcripts		Chimeric Transcripts		Alternative splicing	SNP
ID	Tissue			Number	Novel- Transcripts	Number	COL7A1-UCN2 TIC	Number	
1	Tumor a	51,217,028	17,083	24,932	665	55	+	110,548	112,177
	Normal b	51,478,578	17,010	24,521	624	33	–	85,635	112,710
2	Tumor c	51,098,090	17,278	25,427	789	42	–	108,219	120,160
	Normal d	51,157,672	16,901	24,108	540	34	–	85,393	100,871
3	Tumor e	50,846,034	18,089	26,379	1194	32	+	105,866	134,840
	Normal f	51,363,004	17,590	25,339	1005	26	–	103,312	164,495

### Landscapes of the TIC genome in LC tissues

**Fig. 1:**
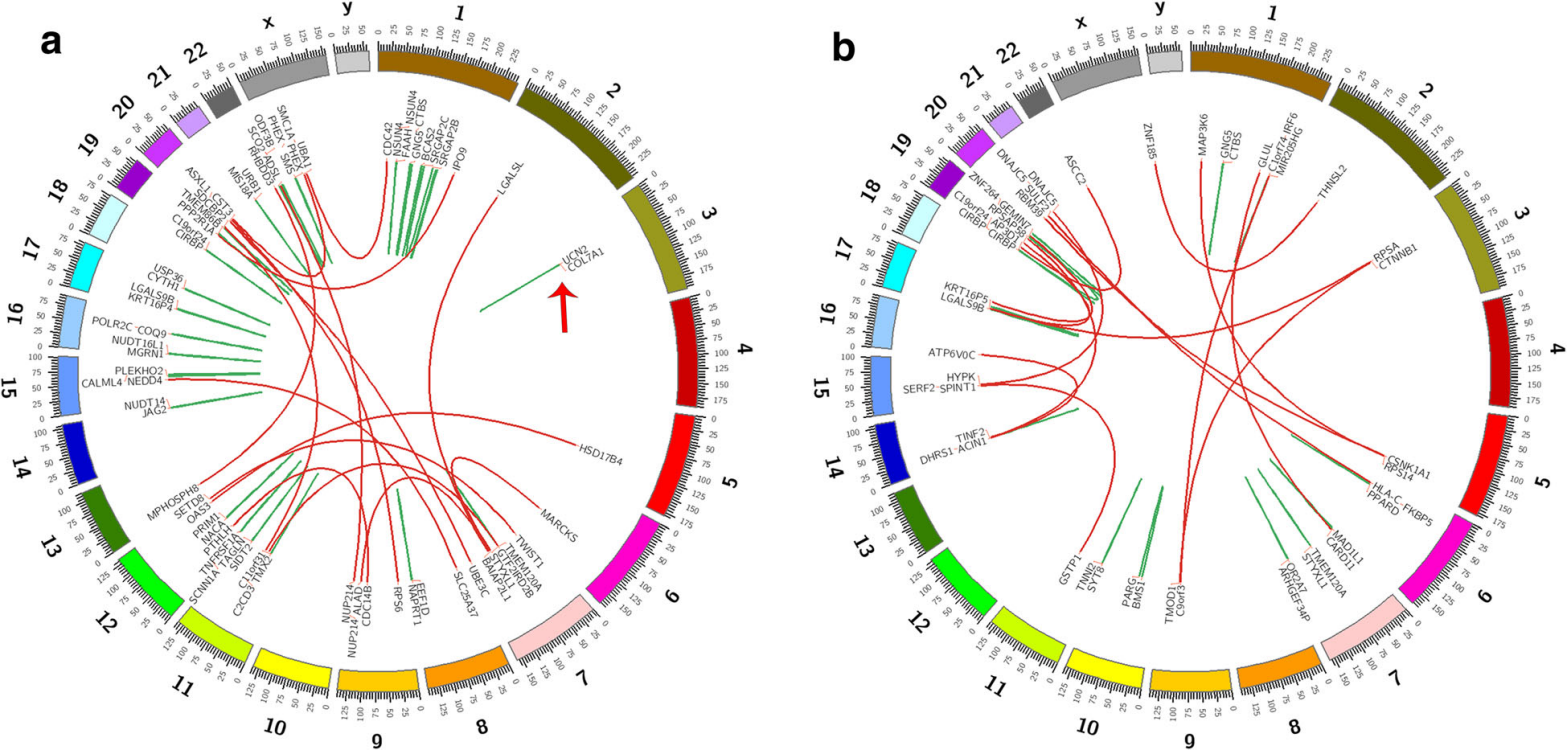
Gene fusion landscape in paired LC and ANMMT samples (**a**) and (**b**) are respective paired samples from one LC patients subjected to transcriptomic analysis. Intrachromosomal and interchromosomal chimeras in the central part of curve lines are marked in red and green, respectively. *COL7A1-UCN2* is shown in (**a**) (red arrows)

In the comparative transcriptome analysis of the three paired LC and ANMMT samples with distinct patterns of tumor differentiation, we identified 87 TICs. We detected the novel chimeric transcript fusion *COL7A1*-*UCN2* in two of the three LC samples but not in their paired ANMMT samples. Also, we did find a coding frameshift in this TIC (Fig. 1 and Additional file 1: Figure S1; Table 4).

**Table 4:** Selected chimeras (10 out of 87 total) identified in three LC samples subjected to transcriptomic analysis

Category	Up-stream Gene	Up-genome position	Down-stream Gene	Down-genome position	Frameshift
Interchromosomal	ALAD	chr9: 116149551	LAMTOR4	chr7: 99751605	NA
Interchromosomal	ARHGAP8	chr22: 45221411	NUP214	chr9: 134103680	frame-shift
Interchromosomal	ASXL1	chr20: 31024580	RPS6	chr9: 19378798	frame-shift
Interchromosomal	BAIAP2L1	chr7: 97984354	MARCKS	chr6: 114180859	frame-shift
Interchromosomal	C2CD3	chr11: 73840431	SMOX	chr20: 4101628	NA
Interchromosomal	CDC14B	chr9: 99284788	PTHLH	chr12: 28116703	frame-shift
Interchromosomal	PPP2R1A	chr19: 52714563	IPO9	chr1: 201827608	frame-shift
Interchromosomal	CST3	chr20: 23618257	UBE3C	chr7: 157060279	frame-shift
Interchromosomal	HSD17B4	chr5: 118809616	OAS3	chr12: 113410220	NA
Interchromosomal	KMT2E	chr7: 104654982	LGALSL	chr2: 64685419	frame-shift
Intrachromosomal	COL7A1	chr3: 48602216	UCN2	chr3: 48600569	frame-shift
Intrachromosomal	COQ9	chr16: 57494016	POLR2C	chr16: 57496472	frame-shift
Intrachromosomal	DENND2C	chr1: 115128157	BCAS2	chr1: 115124012	NA
intrachromosomal	EEF1D	chr8: 144661949	NAPRT1	chr8: 144660113	NA
Intrachromosomal	GBP3	chr1:89474630	CCBL2	chr1: 89435150	frame-shift
Intrachromosomal	HDGF	chr1: 156713444	MRPL24	chr1: 156711380	frame-shift
Intrachromosomal	KRT16P4	chr17: 18354079	LGALS9C	chr17:18387189	NA
Intrachromosomal	MAP2K5	chr15: 68065090	SKOR1	chr15: 68118274	frame-shift
Intrachromosomal	MGRN1	chr16: 4733933	NUDT16L1	chr16: 4744959	frame-shift
Intrachromosomal	NSUN4	chr1: 46826500	FAAH	chr1: 46867763	frame-shift
Intrachromosomal	NUDT14	chr14: 105642871	JAG2	chr14: 105624100	frame-shift
Intrachromosomal	ODF3B	chr22: 50968332	SCO2	chr22: 50962852	NA

**Fig. 2:**
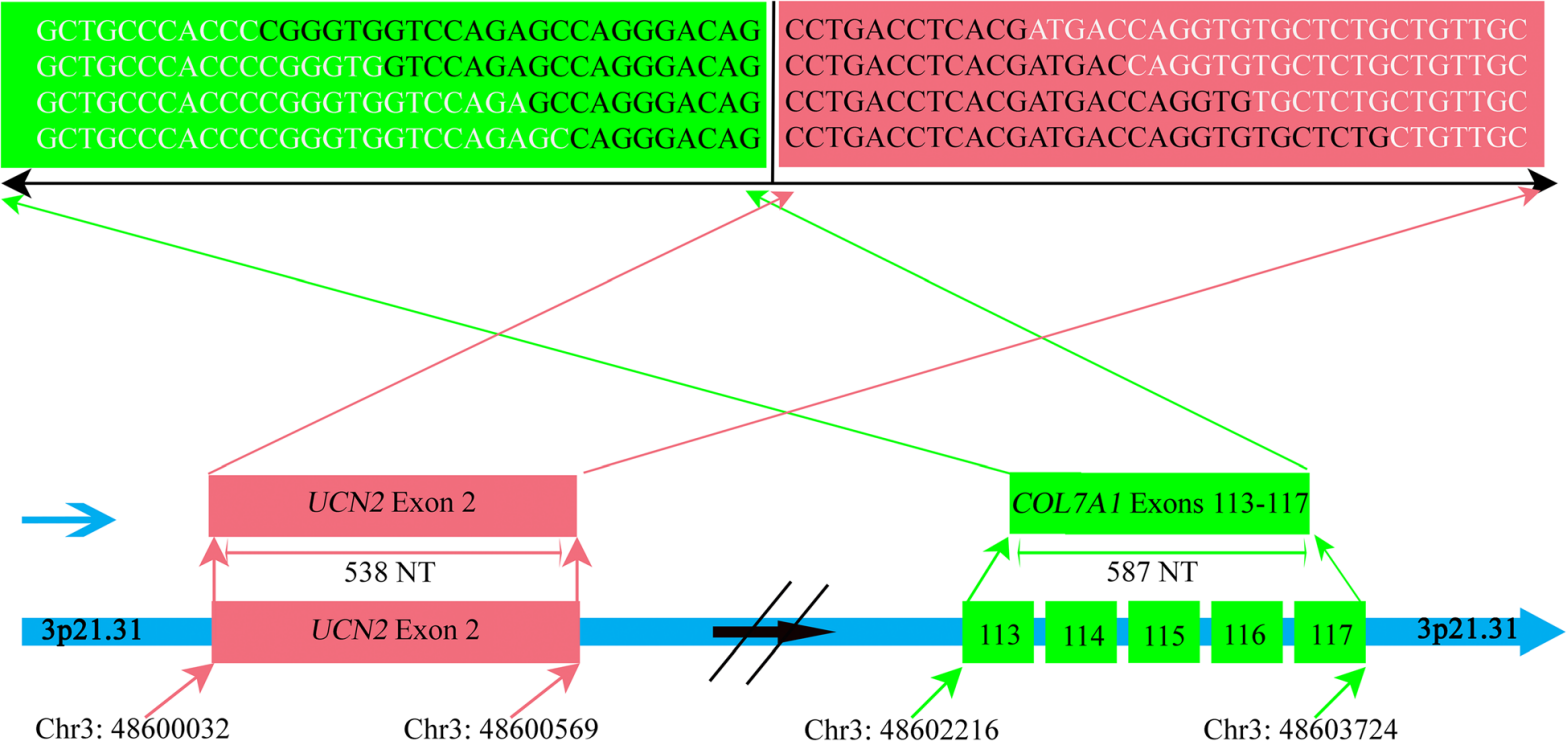
TIC *COL7A1-UCN2* genome landscape in LC

Both the *COL7A1* and *UCN2* genes are located at 3p21.3. In *COL7A1-UCN2*, *COL7A1* is located at exons 113–117 (from Chr. 3: 48602216 to Chr. 3: 48603724) and is 587 nt long. *UCN2* is located at exon 2 (from Chr. 3: 48600032 to Chr. 3:48600569) and is 538 nt long. In *COL7A1-UCN2,* the exon 2 sequence of *UCN2* was frameshifted during the transcript fusion process (Fig. 2).

### COL7A1-UCN2 cDNA validation

**Fig. 3:**
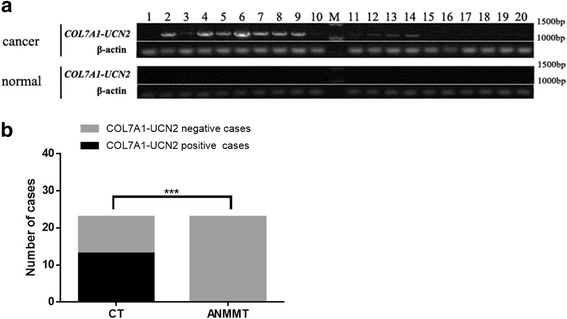
RT-PCR validation of expression of TIC *COL7A1-UCN2 c*DNA in LC and ANMMT samples

In the 20 other tissue sample pairs, RT-PCR analysis revealed *COL7A1-UCN2* cDNA expression in eleven of the LC samples but no TIC transcripts in the ANMMT samples (Fig. 3a). Thus, in this study of 23 LC patients, we detected *COL7A1-UCN2* in 13 patients (57%), and a comparison of the positive TIC distribution in the LC and ANMMT samples demonstrated that positive LC samples were statistically significantly more common than positive ANMMT samples (*p* < 0.0001) (Fig. 3b).

### Expression of COL7A1 and UCN2 mRNA

**Fig. 4:**
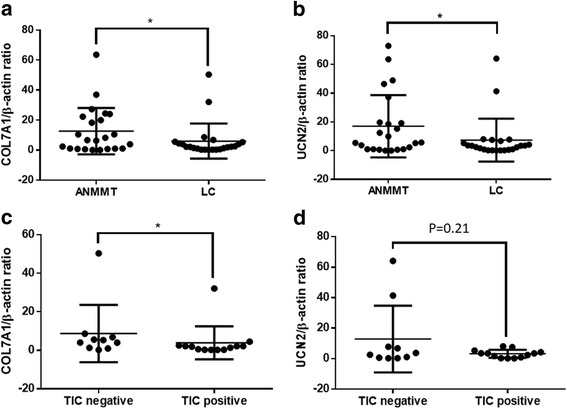
Comparison of *COL7A1* and *UCN2* mRNA expression. LC, laryngeal cancer tissue; ANMMT, adjacent normal membrane mucous Tissue. (**a**) and (**b**) are comparison between LC and ANMMT samples; (**c**) and (**d**) are comparison between TIC *COL7A1-UCN2* negativity and positivity LC samples. *p < 0.05

Among all 23 LC patients, the *COL7A1*:β-actin ratio in the ANMMT samples (12.61 ± 15.52) was significantly higher than that in the LC samples (5.99 ± 11.68; *p* = 0.028) (Fig. 4a). Likewise, the *UCN2*:β-actin ratio in the ANMMT samples (17.02 ± 21.69) was significantly higher than that in the LC samples (7.34 ± 14.90; *p* = 0.021) (Fig. 4b). Furthermore, among all 23 LC tissues, the *COL7A1*:β-actin ratio in the *COL7A1-UCN2* TIC-positive samples (3.89 ± 8.56) was significantly lower than that in the *COL7A1-UCN2* TIC-negative samples (8.71 ± 14.87; *p* = 0.019) (Fig. 4c); likewise, the *UCN2*:β-actin ratio in *COL7A1-UCN2* TIC-positive samples (3.17 ± 2.62) was also lower than that in the *COL7A1-UCN2* TIC-negative samples (12.84 ± 21.85; *p* = 0.21) (Fig. 4d).

### Disrupted coding regions of both COL7A1 and UCN2 in COL7A1-UCN2

**Fig. 5:**
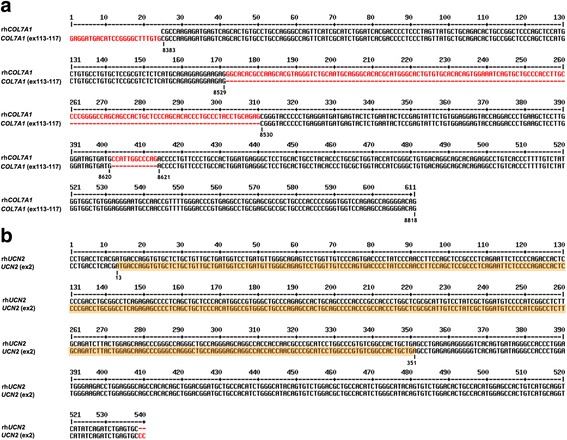
Comparison of DNA sequences between recurrent-hybrid genes and normal genes. **a** Comparison of DNA sequences in recurrent-hybrid *COL7A1* (rh*COL7A1*; the sequence of *COL7A1* in *COL7A1-UCN2*) and *COL7A1* (from exon 113 to exon 117 in a normal *COL7A1* gene). 8383, 8529, 8530, 8620, 8621, and 8818 are the gene sequence numbers in the encoding protein sequence. **b** Comparison of DNA sequences in recurrent-hybrid *UCN2* (rh*UCN2*; the sequence of *UCN2* in *COL7A1-UCN2*) and *UCN2* (nucleotides 1–540 of exon 2 in a normal *UCN2* gene). The gene sequences encoding for the final protein sequence**s** are highlighted in orange. The consensus and inconsistent sequences are shown in black and red, respectively

We compared the DNA sequences in the recurrent hybrid *COL7A1* (rh*COL7A1*, the sequence of *COL7A1* in *COL7A1-UCN2*) and *COL7A1.* The *rhCOL7A1* is located from exon 113 to exon 117 in a normal *COL7A1* gene (Fig. 5a). Besides, the DNA sequences in recurrent hybrid *UCN2* (rh*UCN2*; the sequence of *UCN2* in *COL7A1-UCN2*) and *UCN2* were also compared. The rh*UCN2* was composed of reversed nucleotides 1–540 of exon 2 in a normal *UCN2* gene (Fig. 5b).

**Fig. 6:**
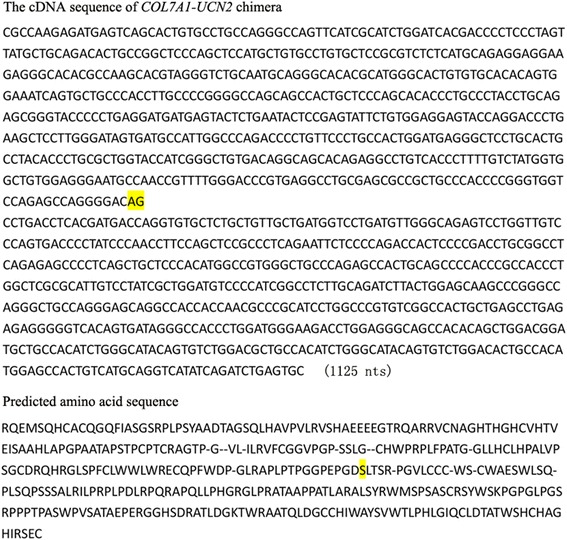
The *COL7A1-UCN2* cDNA sequence and its predicted amino acid sequence. AG (highlighted in yellow) represents the last two nucleotides of *COL7A1*, which may translate into S (Serine, also highlighted in yellow) with the first nucleotide of *UCN2*

From the above, we found the *COL7A1-UCN2* cDNA sequence and its predicted amino acid sequence, in which AG (highlighted in yellow) represents the last two nucleotides of *COL7A1*, which may translate into S (a serine amino acid, also highlighted in yellow), the first nucleotide of *UCN2* (Fig. 6). Based on the above prediction, both the COL7A1 and UCN2 coding regions of *COL7A1-UCN2* were disrupted.

### Effect of COL7A1-UCN2 expression on overall survival in patients with LC

**Fig. 7:**
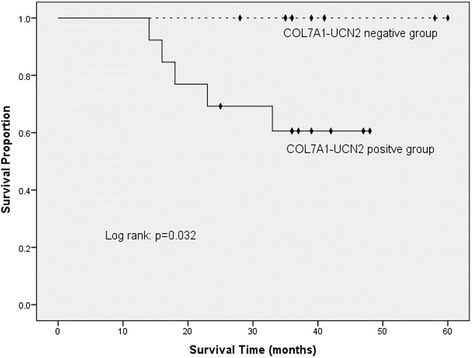
Effect of *COL7A1-UCN2* expression on overall survival in patients positive and negative for this TIC

A Kaplan-Meier analysis revealed that LC patients who were positive for *COL7A1-UCN2* had a significantly worse overall survival time than those patients who were negative did (*p* = 0.032 [log-rank test]) (Fig. 7). Multivariable analysis demonstrated a significant association between *COL7A1-UCN2* expression and overall survival (hazard ratio, 13.2 [95% confidence interval, 1.2–149.5]).

## Discussion

High-throughput transcriptome sequencing provides sufficient information with which to identify candidate oncogenic mRNA chimeras. These chimeric isoforms are usually generated by AS, which is a fundamental mechanism of transcript diversity generation [26–31]. AS generated the TIC *COL7A1-UCN2* between neighboring genes, which is referred to as a read-through event [32]. In the present study, we found *COL7A1-UCN2* positivity in 13 of 23 LC samples, whereas all 23 paired ANMMT samples were negative. This TIC was generated via alternative splicing in the cells of LC tissues. Furthermore, those LC tissues with *COL7A1-UCN2* positivity had lower levels of *COL7A1* and *UCN2* mRNAs as compared to negative LC tissues. Therefore, this TIC potentially down-regulated the expression of the *COL7A1* and *UCN2* genes during and after chimera fusion; and it is thereby associated with poor clinical prognosis because both *COL7A1* and *UCN2* possessed explicit suppressor roles in tumor EMT regulation.

In a previous study, low or nonexistent *COL7A1* expression was associated with the loss of the membrane basement, a specific extracellular matrix (ECM) component, and the promotion of the EMT process in cutaneous squamous cells (CSCCs) [33]. *COL7A1*-produced type VII collagen (ColVII) is the primary component of anchoring fibril protein, which constructs the membrane basement that separates the epithelium from the stroma in epithelial and mucous cells. Invasive epithelial-mucous tumors can be distinguished from benign and pre-invasive lesions by the consistent loss of the surrounding linear basement membrane in a wide variety tissues [33–39]. The breakdown of the basement membrane is a critical early step in EMT, in which oncogenic derivatives of epithelial stem cells are thought to act as intrinsic cancer stem cells that disrupt the basement membrane via the secretion of matrix metalloproteinases (MMPs) [33]. In CSCCs, tumor cells with *COL7A1* knockdown manifested increased migration and higher invasiveness, accompanied by the alteration of EMT marker expression (the decreased expression of E-cadherin and the increased expression of MMP2 and vimentin). Furthermore, ColVII knockdown can decrease epithelial cancer cell differentiation and increase the expression of the chemokine ligand receptors CXCL10-CXCR3 and PLC-β4, which can further facilitate EMT and increase tumor invasion through an autocrine forward loop [22].

In our present study, *COL7A1 mRNA* levels were down-regulated in cancer tissues, and the *COL7A1-UCN2* chimera generation mechanism circumvented TGF-β1’s tumor-suppressive effects and thereby promoted tumor invasion and proliferation. TGF-β1 maintained normal tissue homeostasis and could both suppress and promote tumor proliferation in a time- and concentration-dependent manner [20, 40, 41]. Within this homeostasis, TGF-β1 broadly controlled the ECM, providing transcription regulation for the following genes: *COL1A1, COL1A2, COL3A1, COL5A2, COL6A1, COL6A3, COL7A1,* etc. The ECM is a dense latticework of collagen and elastin that serves as a selective macromolecular filter, it plays a role in mitogenesis and differentiation [42, 43]. Therefore, abnormal ECM homeostasis is a hallmark of cancer. It may be associated with the dysregulation of various collagens and increased tumor invasion because *COL7A1-*produced collagen VII is an essential component of various collagens [20, 43]. TGF-β1 can up-regulate collagen VII in tissues given normal homeostasis, a high concentration, and long-term exposure to TGF-β1 [42]. Collagen VII was found to be down-regulated in cancer tissues, and homeostasis was lost through epigenetic transcription regulation [44], canonical pathway inactivation in TGF-β1 (i.e., TGFR mutation) in cancer cells [45], or ECM alteration in the tumor microenvironment [46]. In our study, we found that cancer tissues had significantly decreased *COL7A1* mRNA levels as compared to paired normal tissues, and we also found that cancer tissues with *COL7A1-UCN2* chimera positivity had significantly lower *COL7A1* mRNA levels than the cancer tissues with *COL7A1-UCN2* chimera negativity. These results might support that the *COL7A1-UCN2* chimera generation mechanism may be associated with the down-regulation of *COL7A1* mRNA*,* which is reflected the degree of invasiveness found in tumor cells.

The activation of the UCN2/corticotropin-releasing factor receptor 2 (CRFR2) axis signaling can inhibit tumor vascularization, cell proliferation and invasion, and EMT [21, 47], whereas the mechanism of *COL7A1-UCN2* chimera generation can potentially down-regulate *UCN2* mRNA and thereby cause the loss of its tumor suppressor role. Both UCN2 and CRFR2 belong to the CRH family, which is known to contain the principal neuroendocrine regulators of stress response in the central nervous system [21, 47, 48]. However, previous studies found that the dysregulation of UCN2/CRFR2 signaling was associated with prostate cancer [49], non-small cell lung carcinoma [50], colorectal cancer (CRC) [21], Lewis lung carcinoma (LLC) [47], and human adrenal and ovarian tumors [51]. Specifically, in vivo and in vitro studies found that UCN2/CRFR2 activation inhibited tumor vascularization and cell proliferation and invasion [21, 47]. Furthermore, in CRC cell lines, the blockage of the UCN2/CRFR2 axis promoted EMT (the altered expression of EMT marker, decreased vimentin, and increased E-cadherin and glycogen synthase kinase 3β expression) via persistent interleukin-6/Stat3 signaling (colonic inflammation regulation) [21].

The coding regions of both COL7A1 and UCN2 were disrupted or destroyed in *COL7A1-UCN2,* and this TIC did not encode a fusion protein. COL7A1 protein includes a Kunitz domain, the deactivation of which induces tumorigenesis [52]. In the rh*COL7A1* coding region, the Kunitz domain is the first 49 residues in the predicted amino acid sequence of *COL7A1-UCN2*, whereas the remaining 96 residues of the Kunitz domain may be disrupted by *UCN2* sequence insertion. In the rh*UCN2* coding region, *UCN2* was frame-shifted, and a discontinuous sequence in the coding region may also disrupt normal UCN2 expression, although *COL7A1-UCN2* includes the complete nucleotides for encoding UCN2 (13–351 nt; 112 amino acids) (Figs. 5 and 6). Therefore, in line with the results of a previous study [14], *COL7A1-UCN2* produced no fusion proteins or independent transcripts.

The presence of *COL7A1-UCN2* in LCs was not the result of stochastic processes. Instead, it was a reflection of DNA damage to a severe degree, and thus it may be associated with poor prognosis. First, we found *COL7A1-UCN2* positivity in 13 of 23 LC samples, whereas all 23 paired ANMMT samples were negative. Second, we found consistent, precise RNA junctions in every recurrent validation in all *COL7A1-UCN2*-positive patient samples. Third, highly expressed genes did not generate TICs randomly. Fourth, a Kaplan-Meier analysis revealed that patients who were positive for *COL7A1-UCN2* had significantly worse overall survival times than did those who were negative.

This study had certain limitations. To validate the DNA rearrangements in chromosomes, the use of a standard fluorescence in situ hybridization (FISH) assay necessitated a minimum distance between the two fused genes (100–150 kb) [53], but the distance between the adjacent ends of *COL7A1* and *UCN2* is less than 20 kb. Thus, we only used long-range RT-PCR to detect the occurrence of *COL7A1-UCN2* cDNA expression in the LC samples. Also, in the AS events, whether the intrinsic TIC-generation mechanism occurs via *cis*-splicing or *trans*-splicing remains unknown [29]. Determining whether TICs function as noncoding RNAs or regulatory RNAs in cancer cell lines without protein participation requires further in vitro evidence. Finally, although our patient sample size was small and potential selection bias could exist, our findings on *COL7A1-UCN2* TIC may provide some novel information to help generate new hypothesis for our future study.

## Conclusion

Our results indicated that the TIC *COL7A1-UCN2* is highly common and enriched in LC samples and that its expression may be associated with LC-cell transition, EMT promotion, and poor LC prognosis. Although its intrinsic generation mechanisms remain largely unknown, *COL7A1-UCN2* may serve as a diagnostic biomarker for early the detection of LC, as well as LC prognosis.

## Additional file

Additional file 1: **Figure S1.** Gene fusion landscape in the other 2 paired LC and ANMMT samples. **c** and **d**, and **e** and **f** are respective paired samples from the other 2 LC patients subjected to transcriptomic analysis (**a** and **b** are in Fig. 1). Intrachromosomal and interchromosomal chimeras in the central part of curve lines are marked in red and green, respectively. *COL7A1-UCN2* is shown in **e** (red arrows). (TIFF 1477 kb)

## Abbreviations

ANMMT: Adjacent normal mucous membrane tissue
AS: Alternative splicing
ColVII: Type VII collagen
CRC: Colorectal cancer
CRFR2: Corticotropin-releasing factor receptor 2
ECM: Extracellular matrix
EMT: Epithelial-mesenchymal transition
FISH: Fluorescence in situ hybridization
LC: Laryngeal cancer
LLC: Lewis lung carcinoma
MMPs: Matrix metalloproteinases
RPKM: Reads per kilobase exon model per million reads
RT-PCR: Real-time polymerase chain reaction
TIC: Transcription-induced chimera
UCN2: Urocortin 2
