# Supplementary figures and images for: Identification of novel enriched recurrent chimeric COL7A1-UCN2 in human laryngeal cancer samples using deep sequencing
Source: BMC Cancer. 2018 Mar 2;18:248. doi: 10.1186/s12885-018-4161-8 (PMC5834868; doi:10.1186/s12885-018-4161-8)

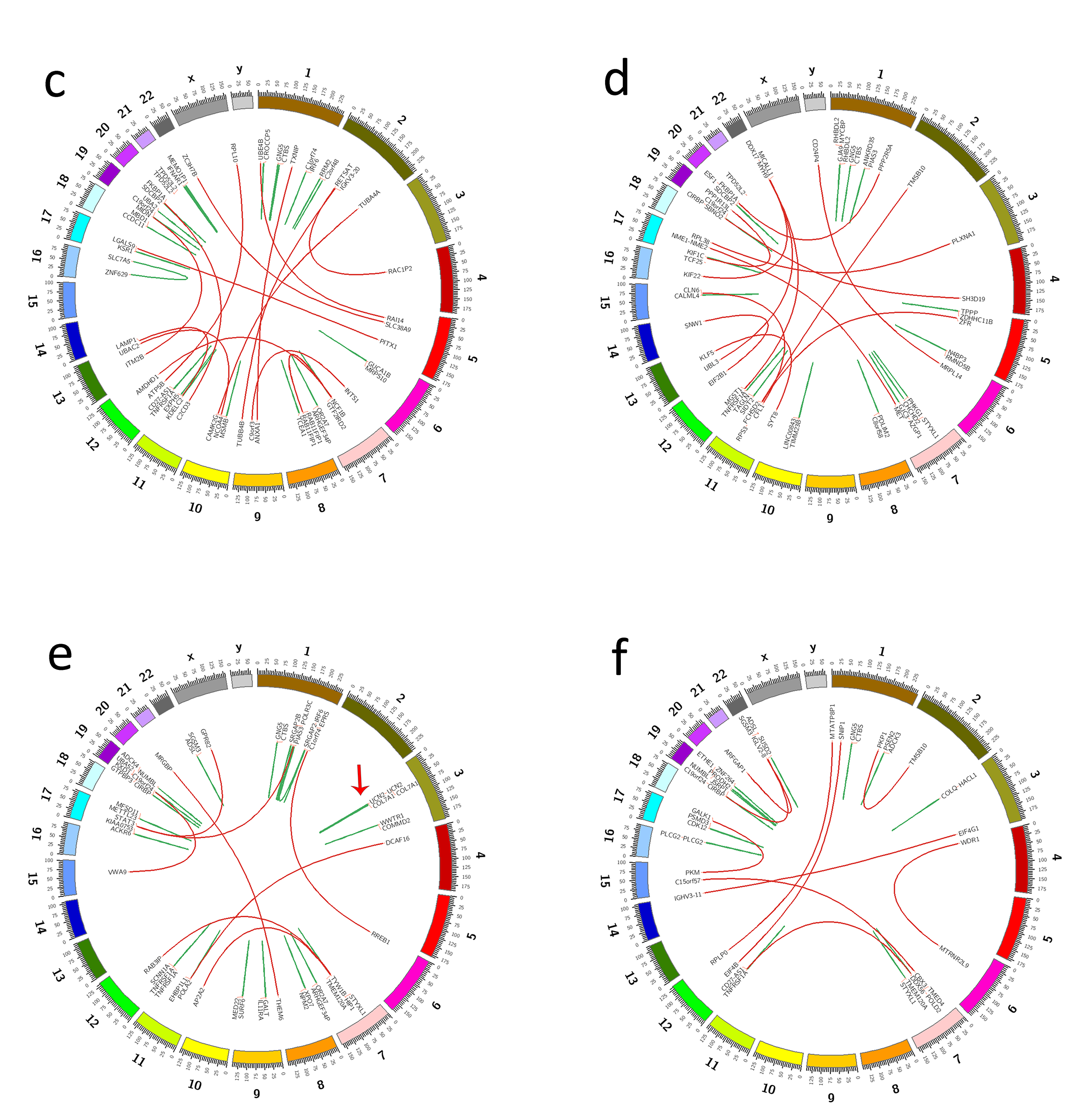

Supplement: Supplementary file 1 — Figure S1. Gene fusion landscape in the other 2 paired LC and ANMMT samples. c and d, and e and f are respective paired samples from the other 2 LC patients subjected to transcriptomic analysis (a and b are in Fig. 1). Intrachromosomal and interchromosomal chimeras in the central part of curve lines are marked in red and green, respectively. COL7A1-UCN2 is shown in e (red arrows). (TIFF 1477 kb) [file 12885_2018_4161_MOESM1_ESM.tif]
